# Acute Kidney Injury in Hospitalized Patients With Exertional Rhabdomyolysis

**DOI:** 10.1001/jamanetworkopen.2024.27464

**Published:** 2024-08-13

**Authors:** Amir H. Sabouri, Brian Yurgionas, Sara Khorasani, Edward J. Durant, Jafar Kafaie, Yun-Yi Hung, Jeffery G. Klingman, Siamack Nemazie

**Affiliations:** 1 Department of Neurology, The Permanente Medical Group, Walnut Creek, California; 2 Department of Neurology, The Permanente Medical Group, San Francisco, California; 3 Department of Emergency Medicine, The Permanente Medical Group, Manteca, California; 4 Department of Neurology, St Louis University, St Louis, Missouri; 5 Division of Research, Kaiser Permanente Northern California, Oakland; 6 Department of Nephrology, The Permanente Medical Group, Santa Rosa, California

## Abstract

**Importance:**

An association between serum creatine kinase (CK) levels and the risk of kidney failure in patients with exertional rhabdomyolysis (ERM) has been suggested. However, the actual incidence of AKI in hospitalized patients with ERM along with the contributing cofactors that may increase the risk of AKI have rarely been investigated.

**Objectives:**

To examine the incidence of kidney injury in hospitalized patients with ERM and to identify additional cofactors that might contribute to the development of kidney injury in patients with ERM.

**Design, Setting, and Participants:**

This retrospective cohort study was conducted in a diverse community population of patients 18 years or older with ERM who were hospitalized across Kaiser Permanente Northern California between January 1, 2009, and December 31, 2019. Patients were initially identified through electronic screening for all-cause rhabdomyolysis admissions, followed by manual medical record reviews to verify their eligibility for the study. The diagnosis of AKI and chronic kidney disease (CKD) was determined using KDIGO (Kidney Disease Improving Global Outcomes) criteria and confirmed by medical record review. Data analysis was performed from October 1, 2023, to January 31, 2024.

**Exposures:**

History of strenuous physical exercise before hospitalization for ERM.

**Main Outcome and Measures:**

Development of AKI, CKD, and compartment syndrome and number of deaths.

**Results:**

Among 3790 patients hospitalized for rhabdomyolysis between 2009 and 2019 in Kaiser Permanente Northern California, 200 (mean [SD] age, 30.5 [8.5] years; 145 [72.5%] male) were confirmed to have ERM via medical record review. Seventeen patients (8.5%) developed AKI, none developed CKD, 1 (0.5%) developed compartment syndrome, and there were no fatalities. There was no association between serum CK levels and the risk of AKI. However, the risk of AKI was significantly higher in patients with ERM who used nonsteroidal anti-inflammatory drugs (NSAIDs) before admission (11 of 17 with AKI [64.7%] vs 40 of 183 without AKI [21.9%], *P* < .001) or experienced dehydration (9 of 183 without AKI [52.9%] vs 9 of 17 with AKI [4.9%], *P* < .001). This analysis suggests that eliminating preadmission NSAID use and dehydration could reduce the risk of potential AKI in patients with ERM by 92.6% (95% CI, 85.7%-96.1%) in this population.

**Conclusions and Relevance:**

The findings of this cohort study of hospitalized patients with ERM suggest that serum CK elevation alone is insufficient as an indicator of AKI in patients with ERM. Concurrent risk factors, such as NSAID use or dehydration, may be associated with AKI development in patients with ERM.

## Key Points

**Question:**

What is the risk of acute kidney injury (AKI) in hospitalized patients with exertional rhabdomyolysis (ERM), and which factors are associated with its development?

**Findings:**

In this cohort study of 200 hospitalized patients with ERM in Northern California, the AKI incidence was 8.5%. No association was observed between serum creatine kinase levels and AKI development; however, preadmission use of nonsteroidal anti-inflammatory drugs or dehydration was associated with a significantly increased risk of developing AKI.

**Meaning:**

These findings suggest that an elevated creatine kinase level is not a reliable indicator of AKI in hospitalized patients with ERM, but preadmission use of nonsteroidal anti-inflammatory drugs or dehydration appear to be associated with AKI.

## Teaser

This cohort study estimates the incidence of acute kidney injury (AKI) in patients hospitalized with exertional rhabdomyolysis and identifies factors associated with development of AKI in this population.

## Introduction

Rhabdomyolysis, a condition characterized by muscle tissue breakdown, can result in severe complications, such as acute kidney injury (AKI) and chronic kidney disease (CKD), and can be fatal, with mortality rates up to 59%.^1,2^ Studies have shown that exertional rhabdomyolysis (ERM), caused by strenuous physical exertion, had a 10-fold increase in incidence in the US from 2000 to 2019^3^ for unknown reasons, with incidence rates varying from 1 to 31.8 per 100 000 population across different populations.^4,5^

Concurrently, research on the outcomes of ERM, both AKI and CKD, and associated risk factors remains scarce. Elevated serum creatine kinase (CK) levels within the range of 5000 to 40 000 U/L (to convert to microkatals per liter, multiply by 0.0167) are reportedly associated with an increased risk of AKI.^2,6,7^ However, certain studies have reported no significant association between CK and creatinine levels in ERM.^8,9^ A previous study found that the risk of AKI in rhabdomyolysis can exceed 50% and varies by etiology.^2^ However, earlier investigations by Clarkson et al^9^ have suggested a significantly lower risk of AKI in ERM. The risk factors that lead to AKI in association with ERM are largely unknown. In this study, we aim to bridge these gaps by examining both the short-term and long-term outcomes of ERM and identifying predisposing factors to AKI within a broad, diverse cohort in Northern California, with a focus on hospitalized patients who are at higher risk of severe complications.

## Methods

### Study Design

We used a retrospective cohort design for comparative analyses to identify factors that differentiate patients with AKI from those without AKI in a cohort of adult patients aged 18 years from multiethnic backgrounds^10^ who, after presentation at an emergency department (ED), were subsequently admitted with ERM from January 1, 2009, to December 31, 2019, to 21 Kaiser Foundation Hospitals within Kaiser Permanente Northern California, a private, not-for-profit, integrated health system with 4.5 million members. Additional inclusion criteria included having engaged in strenuous physical activity, defined based on patient-reported information as any substantial physical activity occurring within 48 hours before hospitalization, which was verified with meticulous manual medical record review. The study was approved by the Kaiser Permanente Northern California Institutional Review Board with a waiver of the requirement for informed consent because risk was minimal. We adhered to the Strengthening the Reporting of Observational Studies in Epidemiology (STROBE) guidelines. Cases were identified using a combination of *International Classification of Diseases, Ninth Revision* (*ICD-9*) and *International Statistical Classification of Diseases and Related Health Problems, Tenth Revision* (*ICD-10*) diagnosis codes to query electronic medical records. In our system, acute outpatient conditions are referred to the ED before hospital admission. We used regular expressions in SAS software, version 9.4 (SAS Institute Inc) and a natural language processing tool to identify clinical notes with key exercise-related phrases. Search terms combined exercise severity (eg, *intense* and *extreme*) and types (eg, *running* and *weightlifting*). We used *ICD-9* and *ICD-10* codes instead of CK levels to define ERM due to the lack of a universally accepted CK threshold, higher CK levels associated with AKI, and variability in CK levels by ethnicity. All cases identified for inclusion received manual medical record review and were verified for eligibility, history of dark urine, dehydration, and nonsteroidal anti-inflammatory drugs (NSAIDs) and acetaminophen exposure before hospitalization. Dark urine was defined based on the documentation of discolored urine, such as dark, brown, cola-colored, or red. Dehydration was defined based on the documentation of any findings, such as dehydration, nausea, vomiting, diarrhea, presyncope, and orthostatic hypotension, in the medical records, extracted through a manual review of all cases. Manual medical record reviews showed high interrater reliability (κ = 0.95; 95% CI, 0.84-1.00), indicating strong consensus among evaluators. We analyzed NSAID and illicit drug exposure within 48 hours before hospital admission in the ERM cohort, including ibuprofen, naproxen, diclofenac, celecoxib, mefenamic acid, etoricoxib, indomethacin, aspirin, cocaine, methamphetamine, amphetamine, methadone, heroin, and acetaminophen. Acute kidney injury was defined according to KDIGO (Kidney Disease Improving Global Outcomes) guidelines, and CKD was identified by a glomerular filtration rate (GFR) below 60 mL/min/1.73 m^2^. Patients with a history of AKI or end-stage kidney disease were excluded. Patients were also excluded if elevated CK levels could be attributed to trauma (defined as any recent physical injury to the body), significant hyperthermia (characterized by recorded body temperatures >38 °C), electrolyte imbalances, infections, or exposure to illicit drugs that could be myotoxic or nephrotoxic.

### Statistical Analysis

Bivariate comparisons involving categorical variables were performed using χ^2^ or Fisher exact tests. Normally distributed continuous variables were compared using 2-sample *t* tests. Comparisons of nonnormally distributed continuous variables were conducted using Wilcoxon rank sum tests. The small number of AKI cases made a multivariable analysis not feasible. Data analysis was performed from October 1, 2023, to January 31, 2024, using SAS software, version 9.4. Two-sided *P* ≤ .05 was considered statistically significant. The prevented fraction of the disease was the proportion of incidents in the unexposed group that could be prevented by exposure. It was calculated using the conventional method.^11^

## Results

**Figure 1.**
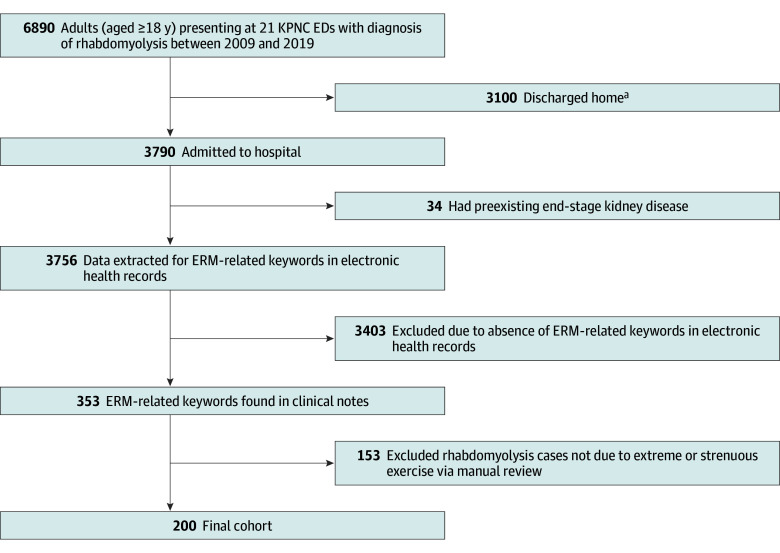
Flow Diagram of Patients With Exertional Rhabdomyolysis (ERM) Eligible for Inclusion in This Study ED indicates emergency department; KPNC, Kaiser Permanente Northern California. ^a^Patients discharged from the emergency department were a heterogeneous group of individuals with rhabdomyolysis due to various causes, including ERM. They were treated according to usual protocols and deemed to have mild disease and be appropriate for discharge by the ED physician.

This retrospective cohort study included patients who presented to the ED and were hospitalized with the primary diagnosis of ERM between January 1, 2009, and December 31, 2019, at Kaiser Permanente Northern California medical centers (21 hospitals), with a mean (SD) follow-up duration of 5.0 (3.4) years. A total of 200 patients (mean [SD] age, 30.9 [8.8] years; 145 [72.5%] male and 55 [27.5%] female; 45 [22.5%] Asian or Pacific Islander, 32 [16.0%] Black or African American, 30 [15.0%] Hispanic or Latino, 86 [43.0%] White, and 7 [3.5%] other, including American Indian or Alaska Native, declined to state, unknown, and any other race) were identified with rhabdomyolysis that was unequivocally attributed to physical exertion and not associated with any other causes (Figure 1). During a 10-year period, the incidence of hospitalized patients with ERM increased from 0.38 to 0.97 cases per 100 000 population, with an overall mean (SD) of 0.63 (2.51) cases per 100 000 population.

Baseline characteristics, laboratory values, treatments, and risks associated with AKI in hospitalized patients with ERM are summarized in Table 1. The CK levels ranged from 1140 to 360 000 U/L (mean [SD], 82 074 [62 978] U/L). In the study cohort, 17 patients developed AKI during hospitalization. The incidence of AKI was 8.5% (95% CI, 5.0%-13.3%). Among the 17 patients with AKI, 7 (41.2%) had stage 1 disease, 1 (5.9%) had stage 2 disease, and 9 (52.9%) had stage 3 disease. Of the patients with stage 3 AKI, 4 did not require dialysis, whereas 5 did. Two of 200 patients (1.0%) were classified as not having a return to baseline creatinine levels (Table 2).

**Table 1.** Baseline Characteristics, Laboratory Values, Treatments, and Risks Associated With AKI in Hospitalized Patients With ERM

Variable	Total (N = 200)	No AKI (n = 183)	AKI (n = 17)	*P* value
Age, mean (SD), y	30.5 (8.5)	30.6 (8.5)	30.1 (8.8)	.81^a^
Gender				
Female	55 (27.5)	53 (29.0)	2 (11.8)	.16^b^
Male	145 (72.5)	130 (71.0)	15 (88.2)	
Race and ethnicity				
Asian or Pacific Islander	45 (22.5)	39 (21.3)	6 (35.3)	.24^b^
Black or African American	32 (16.0)	27 (14.8)	5 (29.4)	
Hispanic or Latino	30 (15.0)	29 (15.8)	1 (5.9)	
White	86 (43.0)	81 (44.3)	5 (29.4)	
Other^c^	7 (3.5)	7 (3.8)	0	
BMI at admission				
<25	61 (30.5)	56 (30.6)	5 (29.4)	.98^d^
25.0-29.9	89 (44.5)	81 (44.3)	8 (47.1)	
≥30	50 (25.0)	46 (25.1)	4 (23.5)	
Charlson Comorbidity Index at admission				
0-1	193 (96.5)	178 (97.3)	15 (88.2)	.11^b^
≥2	7 (3.5)	5 (2.7)	2 (11.8)	
Summer				
No	147 (73.5)	134 (73.2)	13 (76.5)	>.99^b^
Yes	53 (26.5)	49 (26.8)	4 (23.5)	
Diabetes at baseline				
No	196 (98.0)	179 (97.8)	17 (100)	>.99^b^
Yes	4 (2.0)	4 (2.2)	0	
Hypertension at baseline				
No	185 (92.5)	171 (93.4)	14 (82.4)	.12^b^
Yes	15 (7.5)	12 (6.6)	3 (17.6)	
Smoking at baseline				
Yes	16 (8.0)	14 (7.7)	2 (11.8)	.81^b^
Never	154 (77.0)	141 (77.0)	13 (76.5)	
Former smoker	30 (15.0)	28 (15.3)	2 (11.8)	
Ranked Neighborhood Deprivation Index (higher indicates more deprivation)				
0	49 (24.5)	43 (23.5)	6 (35.3)	.73^b^
1	50 (25.0)	46 (25.1)	4 (23.5)	
2	50 (25.0)	47 (25.7)	3 (17.6)	
3	51 (25.5)	47 (25.7)	4 (23.5)	
Dark urine				
No	92 (46.0)	84 (45.9)	8 (47.1)	.93^d^
Yes	108 (54.0)	99 (54.1)	9 (52.9)	
Type of exercise				
Weightlifting	56 (28.0)	54 (29.5)	2 (11.8)	.15^b^
Bootcamp, CrossFit, or interval training	46 (23.0)	42 (23.0)	4 (23.5)	
Extreme vigorous exercise	61 (30.5)	56 (30.6)	5 (29.4)	
Law enforcement, fire academy, or firefighter	25 (12.5)	22 (12.0)	3 (17.7)	
Basketball, football, soccer, tennis, or other sport	12 (6.0)	9 (4.9)	3 (17.7)	
Index hospital LOS, d				
Mean (SD) [range]	3.1 (1.9) [0.0-11.0]	3.0 (1.7) [0.0-11.0]	4.0 (2.9) [1.0-10.0]	.16^a^
Median (IQR)	3.0 (2.0-4.0)	3.0 (2.0-4.0)	3.0 (2.0-6.0)	.26^e^
Furosemide during index hospitalization				
No	187 (93.5)	173 (94.5)	14 (82.4)	.09^b^
Yes	13 (6.5)	10 (5.5)	3 (17.6)	
Mannitol during index hospitalization				
No	197 (98.5)	182 (99.5)	15 (88.2)	.02^b^
Yes	3 (1.5)	1 (0.5)	2 (11.8)	
Bicarbonate during index hospitalization				
No	111 (55.5)	106 (57.9)	5 (29.4)	.04^d^
Yes	89 (44.5)	77 (42.1)	12 (70.6)	
Initial CK during index hospitalization, U/L				
<40 000	74 (37.0)	67 (36.6)	7 (41.2)	.71^d^
≥40 000	126 (63.0)	116 (63.4)	10 (58.8)	
Peak CK during index hospitalization, U/L				
<40 000	63 (31.5)	56 (30.6)	7 (41.2)	.37^d^
≥40 000	137 (68.5)	127 (69.4)	10 (58.8)	
Peak CK during index hospitalization, U/L				
Mean (SD) [range]	81 746.6 (62 829.4) [1149.0-360 000.0]	82 280.0 (61 762.2) [1149.0-360 000.0]	76 004.0 (75 288.5) [4180.0-280 010.0]	.70^a^
Median (IQR)	70 990 (33 600-112 003)	71 589 (33 600-111 013)	47 820 (12 359-124 529)	.37^e^
Initial creatinine during index hospitalization, mg/dL				
<1.1	156 (78.0)	156 (85.2)	0	<.001^d^
1.1-2.5	35 (17.5)	27 (14.8)	8 (47.1)	
>2.5	9 (4.5)	0	9 (52.9)	
Initial creatinine during index hospitalization, mg/dL				
Mean (SD) [range]	1.1 (1.2) [0.5-10.1]	0.9 (0.2) [0.5-1.5]	3.8 (3.0) [1.1-10.1]	.001^a^
Median (IQR)	0.9 (0.8-1.1)	0.9 (0.8-1.0)	2.6 (1.4-4.1)	
Peak creatinine during index hospitalization, mg/dL				
<1.1	153 (76.5)	153 (83.6)		<.001^d^
1.1-2.5	37 (18.5)	30 (16.4)	7 (41.2)	
>2.5	10 (5.0)	0	10 (58.8)	
Peak creatinine during index hospitalization, mg/dL				
Mean (SD) [range]	1.3 (1.8) [0.5-14.1]	0.9 (0.2) [0.5-1.5]	5.3 (4.4) [1.1-14.1]	.001^a^
Median (IQR)	0.9 (0.8-1.1)	0.9 (0.8-1.0)	3.1 (1.4-9.7)	
Initial myoglobin during index hospitalization, μg/L				
Test not performed, No. (%)	185 (92.5)	169 (92.3)	16 (94.1)	NA
Mean (SD) [range]	1078.7 (2384.9) [6.0-9160.0]	1106.7 (2472.4) [6.0-9160.0]	686.0 [686.0-686.0]	
Median (IQR)	104.0 (63.0-686.0)	103.5 (63.0-487.0)	686.0 (686.0-686.0)	
Peak myoglobin during index hospitalization, μg/L				
Test not done, No. (%)	185 (92.5)	169 (92.3)	16 (94.1)	NA
Mean (SD) [range]	1098.7 (2457.7) [6.0-9460.0]	1128.1 (2547.7) [6.0-9460.0]	686.0 [686.0-686.0]	
Median (IQR)	104.0 (63.0-686.0)	103.5 (63.0-487.0)	686.0 (686.0-686.0)	
IVF within 24 h during index hospitalization, mL				
Mean (SD) [range]	6119.6 (2581.6) [0.0-14 000.0]	6061.2 (2505.8) [0.0-140,00.0]	6741.2 (3312.4) [2050.0-13 000.0]	.30^a^
Median (IQR)	6000.0 (4000.0-8000.0)	6000.0 (4000.0-8000.0)	6150.0 (5000.0-9000.0)	
IVF daily during index hospitalization, mL				
Mean (SD) [range]	3741.5 (1709.0) [0.0-9577.7]	3810.0 (1713.0) [0.0-9577.7]	3012.7 (1526.4) [937.9-5479.5]	.07^a^
Median (IQR)	3838.5 (2759.5-4798.1)	3906.4 (2921.6-4810.2)	2821.1 (1469.7-4363.6)	
Preadmission NSAID exposure				
No	149 (74.5)	143 (78.1)	6 (35.3)	<.001^b^
Yes	51 (25.5)	40 (21.9)	11 (64.7)	
Preadmission acetaminophen exposure				
No	193 (92.5)	178 (97.3)	15 (88.2)	.11^b^
Yes	7 (3.5)	5 (2.7)	2 (11.8)	
Preadmission dehydration				
No	182 (91.0)	174 (95.1)	8 (47.1)	<.001^b^
Yes	18 (9.0)	9 (4.9)	9 (52.9)	
Readmission for ERM				
No	192 (96.0)	175 (95.6)	17 (100)	>.99^b^
Yes	8 (4.0)	8 (4.4)	0	

Abbreviations: AKI, acute kidney injury; BMI, body mass index (calculated as weight in kilograms divided by height in meters squared); CK, creatine kinase; ERM, exertional rhabdomyolysis; IVF, intravenous fluid; LOS, length of stay; NA, not applicable; NSAID, nonsteroidal anti-inflammatory drug.

SI conversion factors: To convert CK to microkatals per liter, multiply by 0.0167; creatinine to micromoles per liter, multiply by 88.4; myoglobin to nanomoles per liter, multiply by 0.05814.

^a^ Two-sample *t* test.

^b^ Fisher exact test.

^c^ Includes American Indian or Alaska Native, declined to state, unknown, and any other race.

^d^ χ^2^ test.

^e^ Two-sample Wilcoxon rank sum test.

**Table 2.** Clinical and Paraclinical Characteristics of Patients With Exertional Rhabdomyolysis Who Developed AKI

Age by decade/sex	Clinical presentation	NSAID exposure	Dehydration	Laboratory test results	KDIGO/AKIN AKI staging
20s/Male	Severe muscle cramps in calves and thighs after basketball game, walking difficulty, no dark urine	Yes, ibuprofen, 600 mg 3 times daily	No	Maximum CK, 7492 U/L; maximum Cr, 1.52 mg/dL (normalized in 1 d)	Stage 1
20s/Male	Muscle aches and brown-colored urine and anuria after intense police academy training	Yes, ibuprofen, 800 mg 3 times daily	Yes	Maximum CK, 159 900 U/L; maximum Cr, 8.98 mg/dL (normalized in 66 d)	Stage 3 (required dialysis)
30s/Male	Significant muscle pain, dark-colored urine after intense CrossFit exercise	Yes, ibuprofen (unknown dose)	No	Maximum CK, 280 010 U/L; maximum Cr, 9.75 mg/dL; last Cr, 1.5 mg/dL (not returned to baseline; GFR, >60 mL/min/1.73 m^2^)	Stage 3 (no dialysis)
20s/Male	Muscle pain and dark-colored urine, anuria after weightlifting, nausea, vomiting	None	Yes	Maximum CK, 82 899 U/L; maximum Cr, 10.1 mg/dL (normalized in 16 d)	Stage 3 (no dialysis)
50s/Male	Muscle pain in legs, brown-colored urine after CrossFit	Yes, aspirin (unknown dose)	No	Maximum CK, 46 154 U/L; maximum Cr, 3.1 mg/dL (normalized in 2 d)	Stage 2
20s/Male	Muscle ache, dark-colored urine, poor hydration, nausea, vomiting, diarrhea after intense exercise in fire academy	Yes, ibuprofen (unknown dose)	Yes	Maximum CK, 33 600 U/L; maximum Cr, 10.38 mg/dL (normalized in 24 d)	Stage 3 (required dialysis)
20s/Male	Muscle pain and swelling followed by dark-colored urine, oliguria then symptoms of uremia (drowsiness, abdominal pain, constipation) after intense leg workout	Yes ibuprofen (unknown dose)	No	Maximum CK, 160 000 U/L; maximum Cr, 7.22 mg/dL (normalized in 19 d)	Stage 3 (required dialysis)
30s/Male	Muscle pain, dark-colored urine, oliguria and anuria, after weightlifting	Yes, ibuprofen, 600 mg 3 times daily, and naproxen, 500 twice daily	No	Maximum CK, 143 481 U/L; maximum Cr, 9.74 mg/dL (normalized in 92 d)	Stage 3 (required dialysis including 3 mo as outpatient, has history of amphetamine abuse; resumed exercise 3 mo later; CK increased to 40 000 U/L but no AKI this time)
30s/Male	Muscle soreness, poor hydration, nausea and dizziness, law enforcement, training 8 h/d, discolored urine	None	Yes	Maximum CK, 58 850 U/L; maximum Cr 1.71 mg/dL (normalized in 2 d)	Stage 1
20s/Male	Vomiting for 24 h after 4 h of strenuous exercise, no urine discoloration	None	Yes	Maximum CK, 6621 U/L; maximum Cr, 4.68 mg/dL (normalized in 1397 d)	Stage 3 (no dialysis)
30s/Male	Cramps and pain after playing tennis, no urine discoloration	Yes, ibuprofen (unknown dose)	Yes	Maximum CK, 4180 U/L; maximum Cr, 2.61 (normalized in 1 d)	Stage 3 (no dialysis)
20s/Female	Abdominal pain and severe nausea and vomiting, no urine discoloration	Yes, ibuprofen, 600 mg 3 times daily	Yes	Maximum CK, 38 829; U/L; maximum Cr, 1.37 mg/dL (normalized in 1 d)	Stage 1
20s/Male	Developed presyncope after whole-day exercise without eating or drinking, severe hypovolemia, no urine discoloration	None	Yes	Maximum CK, 12 359 U/L; maximum Cr, 1.94 mg/dL (normalized in 2 d)	Stage 1
20s/Female	Diffuse myalgia after vigorous exercise at bootcamp, vomiting, no urine discoloration	Yes, aspirin, paracetamol, and caffeine (unknown dose)	Yes	Maximum CK, 9679; U/L; maximum Cr, 1.93 (normalized in 3 d)	Stage 1
30s/Male	Abdominal pain, dark urine discoloration after Pilates	None	No	Maximum CK, 47 820 U/L; maximum Cr, 1.38 mg/dL (normalized in 1 d)	Stage 1
20s/Male	Severe back pain, no urine discoloration	Yes, ibuprofen, 600 mg 3 times daily, and naproxen (unknown dose)	No	Maximum CK, 121 172; maximum Cr, 1.34 mg/dL (normalized in 1 d)	Stage 1
30s/Male	Decreased urine output, dark urine, muscle pain, after playing basketball	None	No	Maximum CK, 141 240 U/L; maximum Cr, 8.77 mg/dL (last Cr, 1.5 mg/dL, not returned to baseline; GFR >60 mL/min/1.73 m^2^)	Stage 3 (required dialysis as outpatient for <4 wk)

Abbreviations: AKI, acute kidney injury; AKIN, Acute Kidney Injury Network; CK, creatine kinase; Cr, creatinine; GFR, glomerular filtration rate; KDIGO, Kidney Disease Improving Global Outcomes; NSAID, nonsteroidal anti-inflammatory drug.

**Figure 2.**
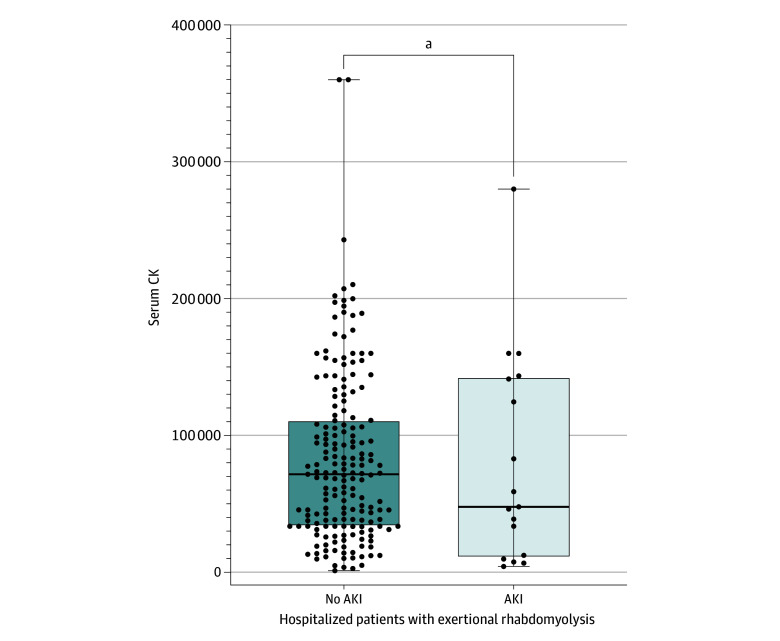
Comparison of Serum Creatine Kinase (CK) Levels in Patients With Exertional Rhabdomyolysis With and Without Acute Kidney Injury (AKI) The ends of the boxes represent the 25th and 75th percentiles, the horizontal line inside the box indicates the median, and the whiskers represent the upper and lower adjacent values. Points that fall beyond the whiskers are shown as dots. ^a^Not significant.

There were no significant differences between patients with ERM who developed AKI and those who did not regarding demographic or clinical variables, including patient’s age, gender, race and ethnicity, baseline body mass index, presence of baseline diabetes, hypertension, smoking, year of disease onset, and season or month of disease occurrence (Table 1). We compared serum CK levels between patients with ERM who developed AKI during hospitalization and those who did not. No significant differences were observed in initial or peak CK levels (Table 1 and Figure 2). Given the reported increased AKI risk with NSAIDs,^12^ we investigated the association between preadmission NSAID and acetaminophen use and AKI development in our cohort. We found a statistically significant association between a history of NSAID exposure and the development of AKI in patients with ERM (11 of 17 patients with AKI [64.7%] vs 40 of 183 patients without AKI [21.9%], *P* < .001) (Table 1). We did not find a statistically significant association between the preadmission use of acetaminophen and the development of AKI. One patient with AKI had a maximum CK level of 4180.0 U/L, which is considered low. However, this patient was exposed to NSAIDs before hospitalization (Table 2), highlighting that CK levels alone may not determine AKI risk. We next examined the association between a history of preadmission dehydration and the risk of AKI development in the cohort. Our findings revealed a significant association between a history of preadmission dehydration and the risk of AKI development (9 of 17 [52.9%] vs 9 of 183 [4.9%], *P* < .001) (Table 1). The findings suggested that if NSAIDs are eliminated, the risk of AKI in patients with ERM could be significantly reduced, with a preventable fraction^11^ of 81.3% (95% CI, 52.1%-92.7%). Furthermore, the elimination of dehydration could reduce the risk by 91.2% (95% CI, 80.0%-96.1%), and addressing both NSAIDs and dehydration could lead to a 92.6% (95% CI, 85.7%-96.1%) preventable fraction. Male patients exhibited a significantly higher prevalence of dark urine than female patients (87 of 145 [60.0%] vs 21 of 55 [38.2%]; *P* = .007) (eTable 1 in Supplement 1). Further analysis revealed that dark urine was associated with significantly higher CK levels across the entire cohort and within both genders (eFigure, eTable 2, and eTable 3 in Supplement 1). We had limited data available for myoglobinuria analysis (15 of 200); however, within the first 24 hours of hospitalization, there was no significant association between dark urine discoloration and urine myoglobin levels, regardless of AKI development or differences in gender.

We did not find significant differences in the types of exercise leading to ERM between 183 patients with ERM who did not develop AKI vs the 17 patients who developed AKI (Table 1). Additionally, we did not observe any significant difference in the length of hospital stay or the rate of daily intravenous fluid use during the index hospitalization between patients with ERM who developed AKI and those who did not, both in the initial 24 hours and in the mean daily intravenous fluid intake throughout the entire hospitalization period. All of the 17 patients with ERM who had AKI in this cohort presented with AKI from the initial laboratory results. There was no statistically significant difference in the intravenous fluid administered within the first 24 hours or during the entire hospitalization between patients with ERM who developed AKI and those who did not. Patients with ERM who developed AKI were significantly more likely to receive mannitol (AKI: 2 [11.8%] vs no AKI: 1 [0.5%]; *P* = .02) and bicarbonate (AKI: 12 [70.6%] vs no AKI: 77 [42.1%]; *P* = .04), but not furosemide, compared with those who did not develop AKI. One patient (0.5%) with ERM developed compartment syndrome and required fasciotomy; however, his kidney function remained normal. There were no fatalities associated with the ERM cases in our cohort. Among the 17 patients who developed AKI, 5 (29.4%) required hemodialysis. Characteristics of the 17 patients with AKI are summarized in Table 2. None of the 17 patients with AKI had prior ERM evaluations. One patient developed ERM without AKI after resuming exercise in 3 months, with a maximum CK level of 143 481 U/L initially (with NSAID exposure) and 40 000 U/L on the second occurrence (without NSAIDs) (Table 2). No patient presented with anemia or thrombocytopenia or had evidence of disseminated intravascular coagulation. We identified only 1 patient with the sickle cell trait, who did not develop AKI. There was no significant difference in the incidence of AKI between patients with ERM admitted in the summer months compared with patients with ERM admitted in other months (Table 1). During our 10-year cohort study, 8 of the 200 patients (4%) were rehospitalized due to ERM but did not develop AKI during either the initial hospitalization or the subsequent episode. There was no difference in the initial or peak CK levels between the patients with ERM who were rehospitalized and those who were not. None of these patients reported a family history of rhabdomyolysis or hereditary myopathy. Genetic testing, encompassing whole exome sequencing and next-generation sequencing, was conducted on 5 of 200 patients with ERM (2.5%), including 3 rehospitalized individuals, to screen for hereditary myopathies; however, no pathogenic mutations were identified.

## Discussion

In this study, we observed an annual ERM incidence of 0.63 per 100 000 individuals, with 8.5% developing AKI. We suggest the actual ERM incidence may be higher due to our focus on hospitalized patients, as subclinical cases go undetected. Conversely, the AKI incidence may be lower than 8.5% because individuals with highly elevated CK levels from strenuous exercise may not develop clinical ERM symptoms.^4,9,13^ Our study suggests that the risk of AKI in ERM is significantly lower compared with other common causes of rhabdomyolysis, such as trauma or sepsis.^2^ These conditions may involve intravascular volume depletion and a plethora of cytokines, which could contribute to kidney injury in the setting of medullary hypoperfusion and elevated serum CK levels.

Our findings suggest that elevated serum CK levels alone may not be a sufficient risk factor for AKI in patients with ERM. For example, cases with CK levels of 360 000 U/L did not result in AKI, whereas levels at 4180 U/L did (Table 2). This finding indicates that AKI development in ERM is influenced by a combination of elevated CK levels and additional risk factors, such as NSAID exposure. Dark urine was associated with male sex in our cohort; however, it was not associated with increased risk of AKI in the entire cohort or for either sex (eTables 1 and 2 in Supplement 1). However, it was associated with significantly higher CK levels across the entire cohort and within each sex (eFigure in Supplement 1). The observed phenomenon can be attributed to potential differences between genders in reporting bias, clearance rates, and symptom onset thresholds, which may be different across genders. Myoglobin, filtered by the kidneys, appears in the urine when plasma concentrations exceed 1.5 mg/dL.^14^ Given the lack of association between dark urine, which could be considered indicative of myoglobinuria, and AKI, this observation may suggest that, similar to serum CK, myoglobinuria alone may not suffice to cause AKI in patients with ERM.

Our study suggests that eliminating key risk factors, such as NSAID use and dehydration, may potentially reduce the incidence of AKI in patients with ERM. Animal studies offer a parallel perspective that elevated CK levels alone may be insufficient to cause AKI in ERM, emphasizing the crucial role of NSAIDs and dehydration.^15,16^ The association of prior dehydration with the development of AKI from rhabdomyolysis was also supported in these models.^15^ Interestingly, a previous study examined the effects of acetaminophen and ibuprofen on kidney function during exercise and dehydration. Ibuprofen was found to have a small but significant association with GFR in a sodium-depleted state, whereas acetaminophen had no such effect.^12^ These findings suggest the synergistic effects of NSAIDs and dehydration during strenuous exercise on kidney function and underscore the importance of choosing acetaminophen over NSAIDs for patients experiencing muscle pain.

The reduced GFR observed with NSAID use is primarily due to the inhibition of kidney prostaglandins, which regulate kidney hemodynamics. NSAIDs inhibit cyclooxygenase, reducing prostaglandin synthesis by 50% to 60%, affecting arteriole diameters and kidney filtration.^2,17^ In 2 of 17 (11.8%) of our patients with ERM and AKI, no history of NSAID ingestion or dehydration was identified. This finding may be due to unreported NSAIDs, other nephrotoxic agents, dehydration, or unknown risk factors. Our findings suggest that straightforward interventions, such as avoiding NSAIDs and prioritizing hydration, may potentially lower AKI risk in those with intense exercise-induced muscle pain. A previous study suggests that more than 50% of healthy volunteers exhibited CK levels in the rhabdomyolysis range after strenuous exercise,^9^ underscoring the significant clinical relevance of our findings.

Finally, our study revealed that none of the patients with ERM who were without AKI at admission developed AKI subsequently; in other words, all patients with ERM diagnosed with AKI on admission exhibited laboratory findings consistent with this diagnosis in their initial evaluation. Similar findings were reported elsewhere by Delaney and Vohra^18^ that a normal first creatinine level was a strong indicator of a normal second creatinine level. The current literature lacks sufficient studies to inform clinicians about treating patients hospitalized for ERM, with minimal consensus on the appropriate timing for discharge.^19^ The median hospital stay for patients without AKI was 3 days, with all treated with fluids. Our findings suggest that hospitalization solely for hydration therapy and serum CK monitoring in most ERM cases may be unnecessary, potentially reducing hospitalizations. Our study concurs with prior research suggesting a lack of a clear discharge CK level threshold for hospitalized patients with ERM and challenges the common practice of extended hospital stays solely for serum CK monitoring until reaching certain low-risk levels.^14,19^ Future studies should emphasize identifying patients at lower risk of developing AKI.

To our knowledge, our study represents the most extensive research to date on patients with ERM, and it has widespread implications across various disciplines. The findings have broad implications, from recreational activities to professional, institutional, and health care settings. These implications encompass physical health–oriented training activities, such as aerobic exercises and indoor cycling sessions. Moreover, these findings are relevant to multiple sectors and disciplines, including fire and police academies as well as military training. Importantly, this study’s implications are broad within the medical field and applicable to multiple specialties. Lastly, the results of this study are crucial for public health educators, broadening our comprehension of the considerable effects that over-the-counter medications may exert on public health.

### Strengths and Limitations

A major strength of our study is the focus on a community-based population with a diverse demographic and wide range of ethnic backgrounds,^10^ in contrast to the predominantly homogenous cohorts featured in the existing literature. Distinguished by its longitudinal design, this study is a pioneer in exploring long-term ERM outcomes, offering insights into the progression of AKI to CKD and its implications.

Our retrospective study faced common challenges, including missing data on NSAID dosage^20^ and urine myoglobin. A notable limitation is potential ascertainment bias, as patients with higher creatinine levels were more likely to be questioned about NSAID exposure and dehydration. Another limitation of our study is defining dehydration through patient-reported symptoms and health care professional examinations, and the lack of a thorough assessment of objective values, such as serum or urine osmolality, due to missing data. We also acknowledge the potential influence of nephrotoxic cofactors, environmental variables, and genetic factors such as sickle cell on AKI development in patients with ERM.^21^ We also acknowledge that because we did not include patients with ERM discharged from the ED in the study, the incidence of AKI-induced ERM may be overestimated. Several questions remain unanswered, including the identification of additional risk factors for AKI in patients with ERM, such as environmental factors, patient behaviors, and genetic predispositions. This study suggests that rhabdomyolysis, as a heterogeneous disease, likely has vastly different outcomes and prognoses, depending on its underlying cause, the target population, and environmental factors. Future research should aim to clarify outcomes related to ERM and focus on the etiologic factors specific to rhabdomyolysis rather than consolidating diverse groups into a single category.

## Conclusions

This large, community-based study significantly advances our understanding of AKI in patients with ERM. It demonstrates a lower AKI risk compared with other causes of rhabdomyolysis and challenges existing assumptions by finding no direct association between elevated CK levels and AKI risk. Additionally, it highlights potential risks associated with NSAID use and dehydration in AKI development and questions the necessity for lengthy hospitalization and associated costs in ERM.

## Notes

Supplement 1. **eTable 1.** Distribution of Dark Urine Based on Gender Among Patients Presenting With Exertional Rhabdomyolysis **eFigure**. Serum CK Levels Based on Gender and Dark Urine **eTable 2.** Association of Dark Urine With Serum CK Levels in Each Gender and the Total Cohort of Patients With Exertional Rhabdomyolysis **eTable 3.** Serum CK Levels Association With Dark Urine

Supplement 2.
Data Sharing Statement

## References

[1] de Meijer AR, Fikkers BG, de Keijzer MH, van Engelen BGM, Drenth JPH. Serum creatine kinase as predictor of clinical course in rhabdomyolysis: a 5-year intensive care survey. Intensive Care Med. 2003;29(7):1121-1125. doi:10.1007/s00134-003-1800-5 12768237

[2] McMahon GM, Zeng X, Waikar SS. A risk prediction score for kidney failure or mortality in rhabdomyolysis. JAMA Intern Med. 2013;173(19):1821-1828. doi:10.1001/jamainternmed.2013.9774 24000014 PMC5152583

[3] Boden BP, Isaacs DJ, Ahmed AE, Anderson SA. Epidemiology of exertional rhabdomyolysis in the United States: analysis of NEISS database 2000 to 2019. Phys Sportsmed. 2022;50(6):486-493. doi:10.1080/00913847.2021.1956288 34278922

[4] Luetmer MT, Boettcher BJ, Franco JM, Reisner JH, Cheville AL, Finnoff JT. Exertional rhabdomyolysis: a retrospective population-based study. Med Sci Sports Exerc. 2020;52(3):608-615. doi:10.1249/MSS.0000000000002178 31652234 PMC8011646

[5] Daniele DO, Murray J. Update: exertional rhabdomyolysis, active component, U.S. Armed Forces, 2017-2021. MSMR. 2022;29(4):15-20. 35608521

[6] Han-Ding M, Xin L, Shu-Yuan L, . Exertional rhabdomyolysis in newly enrolled cadets of a military academy. Muscle Nerve. Published online June 20, 2021. doi:10.1002/mus.27355 34151436

[7] Long B, Koyfman A, Gottlieb M. An evidence-based narrative review of the emergency department evaluation and management of rhabdomyolysis. Am J Emerg Med. 2019;37(3):518-523. doi:10.1016/j.ajem.2018.12.061 30630682

[8] El-Abdellati E, Eyselbergs M, Sirimsi H, . An observational study on rhabdomyolysis in the intensive care unit: exploring its risk factors and main complication: acute kidney injury. Ann Intensive Care. 2013;3(1):8. doi:10.1186/2110-5820-3-8 23497406 PMC3614462

[9] Clarkson PM, Kearns AK, Rouzier P, Rubin R, Thompson PD. Serum creatine kinase levels and renal function measures in exertional muscle damage. Med Sci Sports Exerc. 2006;38(4):623-627. doi:10.1249/01.mss.0000210192.49210.fc 16679975

[10] Marras C, Van den Eeden SK, Fross RD, . Minimum incidence of primary cervical dystonia in a multiethnic health care population. Neurology. 2007;69(7):676-680. doi:10.1212/01.wnl.0000267425.51598.c9 17698789

[11] Hennekens CH, Buring JE. Mayrent, Epidemiology in Medicine. Little Brown and Co; 1987.

[12] Farquhar WB, Morgan AL, Zambraski EJ, Kenney WL. Effects of acetaminophen and ibuprofen on renal function in the stressed kidney. J Appl Physiol (1985). 1999;86(2):598-604. doi:10.1152/jappl.1999.86.2.598 9931196

[13] Kenney K, Landau ME, Gonzalez RS, Hundertmark J, O’Brien K, Campbell WW. Serum creatine kinase after exercise: drawing the line between physiological response and exertional rhabdomyolysis. Muscle Nerve. 2012;45(3):356-362. doi:10.1002/mus.22317 22334169

[14] Brown CVR, Rhee P, Chan L, Evans K, Demetriades D, Velmahos GC. Preventing renal failure in patients with rhabdomyolysis: do bicarbonate and mannitol make a difference? J Trauma. 2004;56(6):1191-1196. doi:10.1097/01.TA.0000130761.78627.10 15211124

[15] Lalich JJ. The influence of in vitro hemoglobin modification on hemoglobinuric nephrosis in rabbits. J Lab Clin Med. 1952;40(1):102-110. 14938754

[16] Bauereiss K, Hofbauer KG, Konrads A, Gross F. Effect of saralasin and serum in myohaemoglobinuric acute renal failure of rats. Clin Sci Mol Med. 1978;54(5):555-560. doi:10.1042/cs0540555 750157

[17] Zambraski EJ, Rofrano TA, Ciccone CD. Effects of aspirin treatment on kidney function in exercising man. Med Sci Sports Exerc. 1982;14(6):419-423. doi:10.1249/00005768-198206000-00002 7162386

[18] Delaney K, Vohra R. Prediction of safe discharge of emergency department patients with acute rhabdomyolysis. Poster presented at 24th International Symposium on Intensive Care and Emergency Medicine; March 30-April 2, 2004; Brussels, Belgium. Accessed May 2, 2024. https://ccforum.biomedcentral.com/articles/10.1186/cc2621

[19] Oh RC, Arter JL, Tiglao SM, Larson SL. Exertional rhabdomyolysis: a case series of 30 hospitalized patients. Mil Med. 2015;180(2):201-207. doi:10.7205/MILMED-D-14-00274 25643388

[20] Pérez Gutthann S, García Rodríguez LA, Raiford DS, Duque Oliart A, Ris Romeu J. Nonsteroidal anti-inflammatory drugs and the risk of hospitalization for acute renal failure. Arch Intern Med. 1996;156(21):2433-2439. doi:10.1001/archinte.156.21.2433 8944736

[21] Ward MM. Factors predictive of acute renal failure in rhabdomyolysis. Arch Intern Med. 1988;148(7):1553-1557. doi:10.1001/archinte.1988.00380070059015 3382301

