# Supplementary material for: Acute Kidney Injury in Hospitalized Patients With Exertional Rhabdomyolysis
Source: JAMA Netw Open. 2024 Aug 13;7(8):e2427464. doi: 10.1001/jamanetworkopen.2024.27464 (PMC11322840; doi:10.1001/jamanetworkopen.2024.27464)
Supplement: Supplement 1. — eTable 1. Distribution of Dark Urine Based on Gender Among Patients Presenting With Exertional Rhabdomyolysis eFigure. Serum CK Levels Based on Gender and Dark Urine eTable 2. Association of Dark Urine With Serum CK Levels in Each Gender and the Total Cohort of Patients With Exertional Rhabdomyolysis eTable 3. Serum CK Levels Association With Dark Urine [file jamanetwopen-e2427464-s001.pdf]

Supplementary Online Content

Sabouri AH, Yurgionas B, Khorasani S, et al. Acute kidney injury in hospitalized patients with exertional rhabdomyolysis. *JAMA Netw Open*. 2024;7(8):e2427464. doi:10.1001/jamanetworkopen.2024.27464

eTable 1. Distribution of Dark Urine Based on Gender Among Patients Presenting With Exertional Rhabdomyolysis

eFigure. Serum CK Levels Based on Gender and Dark Urine

eTable 2. Association of Dark Urine With Serum CK Levels in Each Gender and the Total Cohort of Patients With Exertional Rhabdomyolysis

eTable 3. Serum CK Levels Association With Dark Urine

This supplementary material has been provided by the authors to give readers additional information about their work.

© 2024 Sabouri AH et al. *JAMA Network Open.*

eTable 1: Distribution of Dark Urine Based on Gender Among Patients Presenting with Exertional Rhabdomyolysis

	Total (N=200)	Female (N=55)	Male (N=145)	P Value
**Dark Urine**				**0.007**
0	92 (46.0)	34 (61.8)	58 (40.0)	
1	108 (54.0)	21 (38.2)	87 (60.0)	

eFigure 1: Serum CK levels based on gender and dark urine

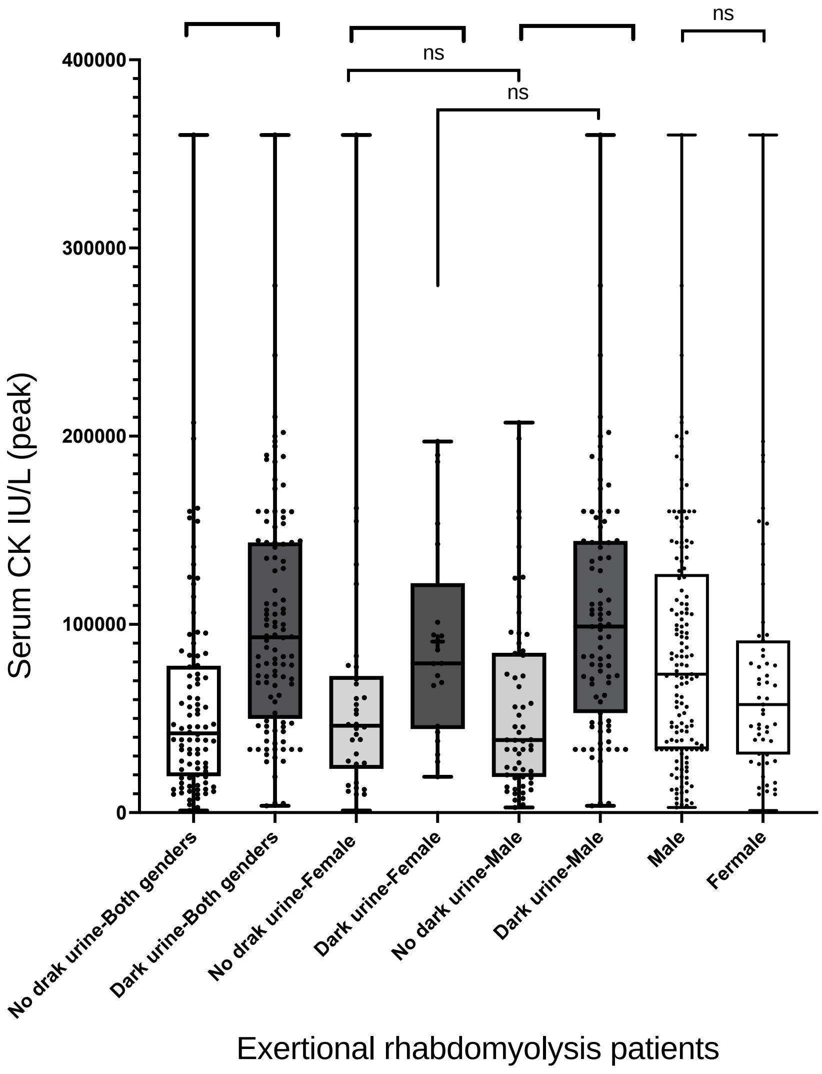

© 2024 Sabouri AH et al. *JAMA Network Open.*

eTable2: Association of Dark Urine with Serum CK Levels in Each Gender and the Total Cohort of Patients with Exertional Rhabdomyolysis

Variables	Total (N=200)	Female + dark urine (N=21)	Female No dark urine (N=34)	Male + dark urine (N=87)	Male No dark urine (N=58)	P Value
**CK initial during index hospitalization**						<.0001
Mean ± SD	71277.8±56608.4	82428.5±51317.5	44439.3±38619.4	93179.4±59808.4	50121.2±48375.3	
Median (IQR)	60787 (30083-99536)	69367 (45273-94443)	31614 (13079-60801)	78693 (46154-129704)	33482 (14793-67763)	
**CK Peak during index hospitalization**						<.0001
Mean ± SD	82074.4±62978.4	90890.2±54130.8	61616.3±66354.3	105018.1±63675.6	56459.7±49022.5	
Median (IQR)	70990 (33600-113840)	79211 (45909-101170)	46147 (25743-70939)	98824 (52880-144305)	38519 (19020-84558)	
**Ck at discharge during index hospitalization**						0.0332
Mean ± SD	24562.6±28485.0	22047.0±16974.2	19528.4±29324.2	31214.5±33170.8	18446.7±21229.2	
Median (IQR)	14724 (6070.5-31063)	16376 (10438-31899)	11691 (5158.0-18818)	19980 (8330.0-41460)	11473 (5220.0-22054)	

eTable3: Serum CK levels association with dark urine

Variables	Total (N=200)	No Dark urine(N=92)	Dark Urine (N=108)	P Value
**CK initial during index hosp**				<.0001
Mean ± SD	71277.8±56608.4	48021.4±44880.8	91088.9±58185.2	
Median (IQR)	60787 (30083-99536)	32994 (13580-67347)	78288 (45714-119457)	
**CK Peak during index hosp**				<.0001
Mean ± SD	82074.4±62978.4	58365.4±55751.4	102271.0±61952.1	
Median (IQR)	70990 (33600-113840)	42066 (19495-77804)	93162 (50800-143537)	
**CK discharge during index hosp**				0.0073
Mean ± SD	24562.6±28485.0	18846.5±24380.4	29431.9±30846.4	
Median (IQR)	14724 (6070.5-31063)	11473 (5189.0-21929)	17486 (9843.5-39639)	

© 2024 Sabouri AH et al. *JAMA Network Open.*
