# Supplementary material for: Acute Kidney Injury in Hospitalized Patients With Exertional Rhabdomyolysis
Source: JAMA Netw Open. 2024 Aug 13;7(8):e2427464. doi: 10.1001/jamanetworkopen.2024.27464 (PMC11322840; doi:10.1001/jamanetworkopen.2024.27464)
Supplement: Supplement 2. — Data Sharing Statement [file jamanetwopen-e2427464-s002.pdf]

Data Sharing Statement

Sabouri. Acute Kidney Injury in Hospitalized Patients With Exertional Rhabdomyolysis. *JAMA Netw Open*. Published August 13, 2024. doi:10.1001/jamanetworkopen.2024.27464

Data
Data available: No

Additional Information
Explanation for why data not available: Data sharing will be depending in my institutions policy
